# Cataract surgery in Southern Ethiopia: distribution, rates and determinants of service provision

**DOI:** 10.1186/1472-6963-13-480

**Published:** 2013-11-19

**Authors:** Esmael Habtamu, Zebiba Eshete, Matthew J Burton

**Affiliations:** 1 International Centre for Eye Health, Faculty of Infectious and Tropical Diseases, London School of Hygiene & Tropical Medicine, London, UK; 2 Department of Ophthalmology, College of Medicine & Health Sciences, Hawassa University, Hawassa, Ethiopia

**Keywords:** Cataract, Cataract surgical rate, Ethiopia, Service provision, Surgeon

## Abstract

**Background:**

Cataract is the leading cause of blindness worldwide, with the greatest burden found in low-income countries. Cataract surgery is a curative and cost-effective intervention. Despite major non-governmental organization (NGO) support, the cataract surgery performed in Southern Region, Ethiopia is currently insufficient to address the need. We analyzed the distribution, productivity, cost and determinants of cataract surgery services.

**Methods:**

Confidential interviews were conducted with all eye surgeons (Ophthalmologists & Non-Physician Cataract Surgeons [NPCS]) in Southern Region using semi-structured questionnaires. Eye care project managers were interviewed using open-ended qualitative questionnaires. All eye units were visited. Information on resources, costs, and the rates and determinants of surgical output were collected.

**Results:**

Cataract surgery provision is uneven across Southern Region: 66% of the units are within 200 km of the regional capital. Surgeon to population ratios varied widely from 1:70,000 in the capital to no service provision in areas containing 7 million people. The Cataract Surgical Rate (CSR) in 2010 was 406 operations/million/year with zonal CSRs ranging between 204 and 1349. Average number of surgeries performed was 374 operations/surgeon/year. Ophthalmologists and NPCS performed a mean of 682 and 280 cataract operations/surgeon/year, respectively (p = 0.03). Resources are underutilized, at 56% of capacity. Community awareness programs were associated with increased activity (p = 0.009). Several factors were associated with increased surgeon productivity (p < 0.05): working for >2 years, working in a NGO/private clinic, working in an urban unit, having a unit manger, conducting outreach programs and a satisfactory work environment. The average cost of cataract surgery in 2010 was US$141.6 (Range: US$37.6–312.6). Units received >70% of their consumables from NGOs. Mangers identified poor staff motivation, community awareness and limited government support as major challenges.

**Conclusions:**

The uneven distribution of infrastructure and personnel, underutilization by the community and inadequate attention and support from the government are limiting cataract surgery service delivery in Southern Ethiopia. Improved human resource management and implementing community-oriented strategies may help increase surgical output and achieve the “Vision 2020: The Right to Sight” targets for treating avoidable blindness.

## Background

In low-income countries, cataract accounts for about half of blindness and has a major impact on poverty [1,2]. It is estimated that in East Africa between 3,000 and 10,000 new cases per million population develop each year [3]. In Ethiopia, adults of 50 years and above constitute 10% of the population [4]. Cataract is the leading cause of blindness and low vision in Ethiopia: >0.5 million people are blind and about 1.2 million are severely visually impaired [5]. Cataract is an age-related condition in which the lens inside the eye becomes opaque, blurring vision. Cataract surgery is a successful, cost-effective intervention [6,7]. The Cataract Surgical Rate (CSR) is the number of cataract operations/million population/year. It is an internationally recognized measure of cataract surgery service activity and an indicator of the availability and acceptability of the service to the population [8,9]. Vision 2020: The Right to Sight, is a World Health Organization (WHO) led global initiative to eliminate avoidable blindness, which recommends a target CSR for Sub-Saharan Africa (SSA) of around 2000 operations/million/year to address the current cataract blindness backlog [9]. However, the productivity of cataract services in most low-income countries is much lower [9,10]. Ethiopia recorded a CSR of 360 in 2006 [11].

Blindness prevention programs are tasked with providing good quality surgery and sustainable services to meet present and future needs [8]. This requires the equitable distribution of resources (human, infrastructure, equipment and material). However, in SSA ophthalmic surgery services are rarely distributed in a manner that corresponds to the population’s need [9,10]. Multiple factors affect productivity of existing cataract surgery services, many of which are provider related, for example, the availability of surgeons, support staff, health facilities, equipment and consumables [12]. The low numbers of patients presenting for surgery might reflect a lack of awareness if programs do not reach out to the community with health-education and publicity [9]. Financial factors are also important determinants [9,13]. However, in Ethiopia, the current cost of surgery from the provider’s perspective is not well characterized.

This study was conducted in the Southern Nations Nationalities and Peoples’ Region (SNNPR). This is the most ethnically diverse, third most populous (16 million people) and geographically largest (112,323 km^2^) region in Ethiopia [4,14]. In SNNPR, ophthalmologists and non-physician cataract surgeons (NPCS) perform cataract surgery. Eye care services have received considerable NGO support in recent years. However, despite this, the number of cataract surgeries performed per year in SNNPR is insufficient to treat the estimated number of incident cataract blind cases (1000 cases/million/year) let alone to reduce the estimated backlog of 130,000 cataract blind persons [5,9]. Ethiopia developed a five year Vision 2020 strategic plan for eye care for the years 2006 to 2010, which is updated every five years [15]. In this strategic plan, SNNPR set itself a target CSR of 900 operations/million/year by 2010 [15]. However, recent reports indicate that there has been little increase: between 2008 and 2009 approximately 6000 surgeries were performed per year (CSR, 375 operations/million/year; Regional Health Bureau Data).

There is a pressing need to improve cataract surgery service delivery in SNNPR, through better utilization and management of available resources. This study was conducted to review the current cataract surgery services in SNNPR, including distribution, productivity and cost. An assessment of cataract surgical outcomes was outside the scope of this study. We believe that the situation in SNNPR reflects cataract surgery services in Ethiopia in general, providing useful information for program planners and service development.

## Methods

### Study participants and health facilities

This study was conducted in SNNPR between June and August 2011. It included all parts of the region where cataract surgery services were delivered and where ophthalmologists and NPCS were based. A complete list of all 16 health facilities where cataract services were being delivered was generated from records of the Regional Health Bureau and supporting NGOs. These were all the units that provide cataract surgery in SNNPR. There were 6 ophthalmologists and 12 NPCS working in SNNPR between January 2010 and August 2011 within government, NGO and private institutions. These were all the available health staff doing cataract surgery in SNNPR. Regardless of current involvement in eye care; each surgeon’s location and contact information were collected. All were contacted by telephone and face-to-face interviews were arranged prior to commencing fieldwork. The number and types of health facilities and the surgeons available to perform cataract surgery were the same in 2010 and 2011. We also contacted and interviewed all three national level program mangers of the NGOs regularly supporting cataract surgery service, the SNNPR Health Bureau Blindness Prevention and Control Program Officer and all seven of the available eye unit managers.

### Data collection

Three separate questionnaires were used to collect data from (a) eye units, (b) surgeons, and (c) program and eye unit managers (See Additional file 1 for data record forms). Questionnaires were pretested with eye units, surgeons and project managers outside SNNPR. Interviews were conducted in Amharic by the investigators (EH & ZE) who travelled to the participant’s place of work.

#### ***Eye unit questionnaire***

All 16 eye units where cataract services were being delivered were visited. Quantitative data were collected using structured questionnaires. The questionnaire had three sections: (1) resources, (2) health facility output and organization, and (3) provider costs. The resources section documented the number and level of staff, equipment and physical facilities. The availability of surgical materials in 2011 was assessed by spot-checking for supplies against a standard required list for cataract surgery. Access to all necessary consumables in 2010 was defined as all consumables being available for at least 2/3 of the year. The output section was completed directly from the unit surgical logbook for 2010 only. This recorded information on the number of cases performed (in the facility and through outreach), numbers of cases operated per session, time allocated to surgery and duration of hospital stay.

#### ***Cost estimates***

The annual provider cost for 2010 was calculated from the following components:

Fixed costs:

• Staff salaries (surgeons, nurses, managers, support staff).

• Equipment (minus 15% for depreciation per year from purchase).

• Utilities (electricity, water).

Variable costs:

• Intraocular lens (IOL).

• Surgical consumables and medication.

• Equipment worth < US$100.

• Patient food and other support.

The number of hours spent each week by all staff on activities related to cataract services, including clinical examinations, surgery and administration in 2010 were estimated. This was pro-rated and the annual salary cost calculated. The calculation included the cost for a single post-operative examination. Information on the purchase price of equipment used to provide the surgical service was provided by the health facilities and supporting NGOs. Where this was not known, the current price of the equipment was used. The cost of transporting equipment to the health facility was included. Where this information was not available, transport was estimated at 10% of the equipment purchase cost. Then, as per WHO recommendation, 15% depreciation was calculated for items with a value of > US$100 and this value was included in the fixed costs estimates [16]. Utility costs were estimated from information provided by the health facility finance department and were included under fixed costs. This was because the eye units did not know the utility costs apportioned to either the eye unit or to cataract surgery services. Therefore, the amount attributable to eye care was estimated at 5%-10% of the total annual cost (depending on the size of the facility). Half of this was allocated to cataract surgery services. Rent was included under utility costs for one private eye unit. Building costs were excluded from the fixed cost analysis, as most government eye units were part of larger health facilities. Variable costs, incurred for each procedure performed, were calculated separately for Extra Capsular Cataract Extraction (ECCE) and Manual Small Incision Cataract Surgery (MSICS).

#### ***Surgeons questionnaire***

A face-to-face confidential interview was conducted with all 18 surgeons working in SNNPR using a semi-structured questionnaire, which included mostly closed, quantitative questions and a few open, qualitative questions. Demographic information was recorded. Data on the individual surgeon’s cataract surgery output in 2010 were collected from their surgical logbooks. Surgeons were asked a range of specific quantitative/closed questions about their training, current surgical practice, working environment, support received, service delivery strategies and factors influencing productivity. We also asked open qualitative questions about what they perceived to be the challenges in delivering the service and their proposed solutions.

A distinction was made between “outreach” and “campaign” services. “Outreach” services are those delivered by eye units regularly to a particular population in health facilities closer to the community, other than the base unit. The term “campaign” was used to refer to an additional service conducted by anybody capable and permitted as a special event, separate from the local fixed or outreach work.

#### ***Managers questionnaire***

In-depth interviews were conducted with 11 managers using open/qualitative questions by a single interviewer (EH) to elicit their opinions on: (1) current cataract surgery service achievements, (2) challenges faced, (3) types of support received/provided, and (4) suggestions on how to develop the service. Responses were recorded as hand written notes in English.

### Analysis

Quantitative data were managed in Access and analyzed using STATA 11. The CSR was calculated using a projected population for 2010, based on the 2007 national population and housing census [4]. Staff numbers and the cost of surgical materials relate to the 2010 period. Productivity and provider unit cost data was analyzed for 2010. Fisher’s exact test was used to test associations between categorical variables. Wilcoxon rank-sum test and Kruskal-Wallis rank test were used for continuous data because of skewed distributions and small numbers. Qualitative data from the managers’ interviews were extracted directly from written notes, major themes coded and presented. Important views are directly quoted.

### Ethics statement

SNNPR Health Bureau Ethics Committee and London School of Hygiene and Tropical Medicine Ethics Committee reviewed and approved this study. Each participant provided written informed consent. This study adhered to the tenets of the Declaration of Helsinki.

## Results

### Distribution of eye care services

**Figure 1:**
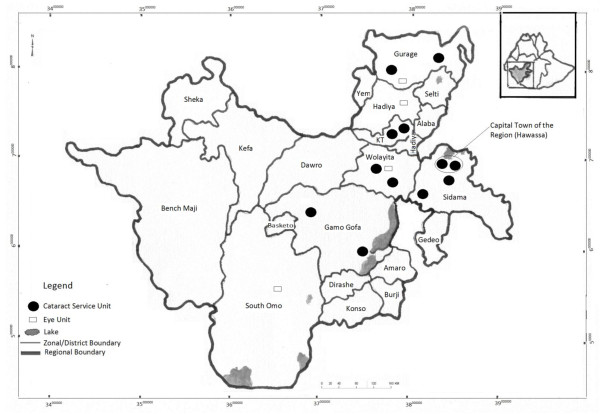
Distribution of units providing cataract surgery in SNNPR in 2010.

There was one tertiary and ten secondary government eye units, four NGO eye units and one private eye clinic. Among these, 12 units provided regular cataract surgery services. Two secondary eye units did not provide surgery in 2010: one had no surgeon and the other was recently established. One NGO refraction unit and another secondary eye unit provided cataract surgery services through a single short campaign during 2010. The geographical distribution of eye units in SNNPR is illustrated in Figure 1. Among the eye units, 10/16 (62.5%) were located in the main urban centers, within 200 km of the regional capital Hawassa. Eight of the 13 zones and an additional eight special districts did not have a cataract surgery unit (Figure 1). All government eye units in SNNPR were established and/or equipped by NGOs.

### Human resources

All six ophthalmologists and 12 NPCS based in SNNPR were interviewed. Their mean age was 36 years (Range: 32–44) and 11 were men. One NPCS was involved in non-eye care private practice because of work placement problems. There were several more midlevel cadres working in eye care: ophthalmic officers, ophthalmic nurses, operating room nurses and integrated eye care workers. On average, four non-surgical staff supported activities in each unit conducting regular cataract surgery. There were seven eye unit managers.

The distribution of human resources between units and across the 13 Zones is shown in Table 1. Overall, there was one surgeon to about one million people. However, the zonal-level surgeon to population ratio varied widely, from 1:70,000 in the regional capital to no services in areas containing about seven million people. Seven surgeons (41.2%) worked within 50 km of the regional capital.

**Table 1:** **Capacity**, **resource utilization**, **and provider cost**

**Zones and eye units**		**Population in millions**	**Surgeons**	**CSR**	**Surgery cases (2010)**	**Eye unit annual capacity and surgeon utilization**				**Cost/operation in USD**		
						***Surgeons***	***Surgeries/surgeon***	***Capacity***	***(Surgeon utilization)***	***Fixed***	***Variable***	***Total***
A. Hawassa town		0.28	5^Ŧ^	-	526							
	Eye unit 1 (Tertiary)ᵻ				777*	3	259	2400	(32.4%)	31.7	30.8	62.5
	Eye unit 2 (Private)ᵻ				200	1	200	800	(25.0%)	53.3	30.4	83.7
B. Zone 1		1.74	3	334	582							
	Eye unit 3				472	2	236	1600	(29.5%)	156.3	30.3	186.6
	Eye unit 4				110	1	110	800	(13.8%)	186.9	49.0	235.9
C. Zone 2		1.67	2	609	1017							
	Eye unit 3				703	1	703	800	(87.9%)	34.6	20.5	55.1
	Eye unit 6				60	1^†^	60	267	(22.5%)	289.8	22.8	312.6
	Eye unit 7				254	0	-	-	-	-	-	-
D. Zone 3		0.74	1	732	542							
	Eye unit 8				107	1^†^	107	267	(40.1%)	204.9	23.9	228.8
	Eye unit 9 (NGO)				435	0	-	-	-	22.7	30.5	53.2
E. Zone 4		1.4	4	1349	1889							
	Eye unit 10 (NGO)				1835	2	918	1600	(114.8%)	7.2	30.4	37.6
	Eye unit 11				54	1^†^	54	267	(20.2%)	256.3	36.2	292.5
	Eye Unit 12				0	1^†^	0	267	(0.0%)	-	-	-
F. Zone 5		3.23	3	435	1404							
	Eye unit 13				1308	3^†^	436	1867	(70.1%)	12.5	30.2	42.7
	Eye unit 14 (NGO)				96	0	-	-	-	82.8	25.5	108.3
G. Zone 6		0.63	0	381	240							
	Eye unit 15				240^‡^	0	-	-	-	-	-	-
H. Zone 7		1.35	0	0	0							
	Eye Unit 16				0	0	-	-	-	-	-	-
I. Zone 8		0.96	0	204	196		-	-	-	-	-	-
J. District 1		0.25	0	1020	255		-	-	-	-	-	-
K. 5 Zones and 7 districts		4.15	0	0	0	0	-	-	-	-	-	-

Variable cost: consumables, medication, *IOL*, food, incentives & equipment with <100 US$ cost. Fixed cost: salaries of personnel involved, equipment with >100US$ cost) & utilities.

ᵻ Provide service to people from other zones, *CSR* includes surgery on people from outside this district. *451 surgeries are performed in Zone 8 and District 1 in outreach program† 5 surgeons only worked for 4 months in 2010 in the eye unit; therefore, 267 operations per surgeon were taken as maximum operation that can be performed within 4 months under favorable conditions. Ŧ including one surgeon not in eye care practice. ‡ 494 surgeries were performed by external surgeons in “campaigns”. These are excluded from capacity and cost analysis. (800 Cataract operations/surgeon are used as maximum capacity).

### Equipment and material resources

Each eye unit estimated the proportion of their surgical supplies that were provided by NGOs; this ranged from 70% to 100%. All but one unit had access to all necessary surgical consumables throughout 2010. The shortage of consumables in a single unit occurred after an NGO suspended its support. All units have an operating room with one or more operating microscopes. All except one unit had at least three cataract surgery sets (median: 4; range: 2–14). All units had a slit lamp microscope (used to examine eyes). One unit did not have an A-scan or keratometer (used to make measurements to choose the intraocular lens (IOL) power). Inpatient beds were available in eight units.

### Surgical activity

During 2010, 10,649 ophthalmic surgeries were performed in SNNPR, of which 6651 (62.5%) were cataract operations. The CSR for 2010 was 406 operations/million/year for the entire region. There was marked variation in CSR between zones where surgery was being performed (range: 204–1349). The region as a whole achieved only 45.3% of the 2010 cataract surgery target of 14,685 operations. The mean number of cataract operations performed per unit was 416 (Median: 220; Range: 0–1835). Most operations were performed at central eye units (4955, [74.5%]); a smaller number of operations were performed through outreach activities (1202, [18.1%]) and campaigns (494, [7.4%]). Nearly all operations (6363/6399 [99%], where data available) involved IOL implantation. Female patients accounted for just under half of the cases operated (3045/6411, [47.5%]), where data is available.

Table 1 compares the capacity and resource utilization of each eye unit in the region. There are no specific standards for the annual number of cataract operations that a single surgeon is expected to perform. Therefore, we used an annual benchmark figure of 800 operations, which we considered feasible, under favorable conditions. Thus, in 2010, SNNPR had the potential to perform 10,935 cataract operations (5 surgeons only worked for 4 months and therefore had the potential capacity of 267 each). However, only 6157 were performed, leading to a regional surgeon utilization level of 56.3%. We excluded 494 surgeries, which were performed in independent campaigns by surgeons coming in from outside Ethiopia, from this analysis of SNNRP service utilization and activity. There were no waiting lists for cataract surgery at any unit in SNNPR. In 2010, five units conducted community cataract awareness activities and two NGO units performed community-based screening. Community awareness programs were associated with a higher surgical output (Wilcoxon rank-sum test, p = 0.009).

### Provider cost and price of cataract surgery

The cost of cataract surgery was estimated for 12 units. Four units were excluded from this cost analysis; no surgery was done at two units during 2010 and at the other two units services were delivered by foreign volunteers in free surgical campaigns. The average provider cost of cataract surgery in 2010 was US$141.6 (Range: US$37.6–312.6), (Table 1). Eye units that did relatively few surgeries had higher fixed costs per operation. The mean variable cost (IOL, consumables and medication) for MSICS was slightly higher (Mean: US$31.2; Range: US$26.5–36.4) than that for ECCE (Mean: US$25.4; Range: US$20.5–30.5). The mean cost of IOL was US$7.2 (Range: US$2.3–12.3). The mean patient charge for cataract surgery was US$31.6 (Median: US$19.8; Range: US$10.0–154.5). On average patients pay US$15.8 (Range: US$10.0–21.7) for cataract surgery in government units compared to US$63.2 (Median: US$33.5; Range: US$31.2–154.5) in private/NGO units (Wilcoxon rank-sum: p = 0.007).

### Surgeon practices and productivity

Among surgeons who were in a position to perform cataract surgery, all six ophthalmologists and nine NPCS were operating. Ophthalmologists had a lower patient visual acuity threshold for operating compared to NPCS. The ophthalmologists all performed MSICS while most NPCS were trained only in ECCE surgery (Table 2). Overall, the average number of operations/surgeon was 374 (Median: 250; Range: 0–1137). Excluding the surgeons who worked for less than the full year, ophthalmologists and NPCS performed a mean of 682 (Median: 662; Range: 250–1137) and 280 (Median: 157; Range: 5–698) cataract operations/year, respectively (Wilcoxon rank-sum test: p = 0.03).

**Table 2:** Practices of ophthalmologists and cataract surgeons

**Variable**		**All (N = 17)**		**Ophthalmologists (N = 6)**		**NPCS (N = 11)**		**P value**
		***n***		***n***		***n***		
Cataract surgeries during training, mean, SD and range (R)		105	SD 39 R 65-200	133	SD 44 R 76-200	90	SD 28 R 65-160	0.03^†^
Years worked as a surgeon, mean, SD and Range (R)		3.4	SD 2.8 R 0.9–10	5.2	SD 4.2 R 1.4–10	2.5	SD 1.1 R 0.9–4.3	0.4^†^
Number of days performing cataract surgery/week: median (range)		1.5	(0–3)	2	(1–3)	1	(0–2)	0.05^†^
Number of surgeries performed/day: median (range)		5	(0–20)	8.5	(2–20)	4.5	(0–15)	_
Working eye unit								0.5^*^
	Government eye unit	14	(82.4%)	4	(66.7%)	10	(90.9%)	
	Private/ NGO	3	(17.6%)	2	(33.3%)	1	(9.1%)	
Visual cut-off point								0.03^*^
	<3/60	12	(70.6%)	2	(33.3%)	10	(90.9%)	
	<6/60	2	(11.8%)	1	(16.7%)	1	(9.1%)	
	<6/36	2	(11.8%)	2	(33.3%)	0	(0%)	
	According to demand	1	(5.9%)	1	(16.7%)	0	(0%)	
Type of surgery^Ŧ^								0.009^*^
	ECCE + PCIOL	8	(47.1%)	0	(0%)	8	(72.7%)	
	MSICS	9	(52.9%)	6	(100%)	3	(27.3%)	
Performing outreach								0.03^*^
	No	12	(70.6%)	2	(33.3%)	10	(90.9%)	
	Yes	5	(29.4%)	4	(66.7%)	1	(9.1%)	
Satisfied by work environment								0.3^*^
	No	7	(41.2%)	1	(16.7%)	6	(54.6%)	
	Yes	10	(58.8%)	5	(83.3%)	5	(45.6%)	

† Wilcoxon rank-sum test, *Fisher’s exact test.

Ŧ Cataract surgeons (NPCS) are trained in ECCE + PCIOL surgery.

Factors potentially influencing productivity, by cadre, are shown in Table 2. Ophthalmologists were more likely to be involved in outreach surgical programs (Fisher’s exact test: p = 0.03). Most ophthalmologists (5/6) were satisfied with their work environment, while less than half of the NPCS (5/11) were satisfied. Ophthalmologists tended to have a longer working experience than NPCS (Mean: 5.2 vs. 2.5 years, p = 0.4). All six ophthalmologists and seven (64%) of the NPCS worked in urban units (in this study rural units were defined as district level secondary eye units).

In a univariate analysis, higher productivity was associated with: longer work experience, work environment satisfaction, working in the NGO sector, working in an urban unit, having a manager, community-based screening and outreach practices (Table 3). Having more than 3 cataract sets did not increase productivity. There is a suggestion that NPCS productivity increases with supervision from or working directly with an ophthalmologist.

**Table 3:** Factors influencing cataract surgery productivity in 2010

**Factor**			**Surgeons N = 17**	**Mean surgery**	**(SD)**	P value*
Demographic						
	Age in years					
		< 35	9	236.4	(263.9)	0.1
		≥ 35	8	527.9	(420.0)	
	Sex					
		Female	6	197.5	(235.3)	0.2
		Male	11	469.6	(398.4)	
Training duration (years)						
	≤ 3		11	205.3	(259.2)	
	4		6	682.2	(345.5)	0.009
Number of surgeries during training						
	< 80		7	115.6	(212.7)	0.02^‡^
	80 – 100		4	478.3	(479.5)	
	>100		6	604.8	(268.2)	
Years worked as a surgeon						
	< 2		5	170.8	(255.6)	0.04^‡^
	2 - 4		8	257	(249.1)	
	4 - 6		1	562	(0)	
	> 6		3	958	(203.3)	
Eye unit type						
	Government eye unit		14	251.1	(257.4)	0.01
	Private/ NGO		3	945.0	(224.6)	
Working location^ᵻ^						
	Rural		4	42.8	(51.2)	0.02
	Urban		13	475.4	(361.8)	
Cataract sets						
	≤ 3		5	356.4	(411.4)	0.8
	≥ 4		12	380.8	(365.9)	
Operating microscope						
	1		7	270.3	(368.0)	0.2
	>1		10	445.9	(367.0)	
Nursing staff						
	≤ 3		6	229.4	(385.1)	0.1
	≥ 4		11	452.4	(348.8)	
Project manager						
	No		5	75	(84.6)	0.03
	Yes		12	492	(368.2)	
Performing outreach						
	No		12	267.9	(356.4)	0.04
	Yes		5	627.2	(273.5)	
Community based screening						
	No		15	301.1	(314.5)	0.05
	Yes		2	917.5	(310.4)	
Satisfied by the work environment						
	No		7	159.6	(204.1)	0.03
	Yes		10	523.4	(388.3)	
Supervision (NPCS only)						
	No		4	75	(89.1)	0.2
	Yes		7	279.7	(299.4)	
Working with ophthalmologist (NPCS only)						
	No		5	36.4	(47.7)	0.07
	Yes		6	346.0	(283.5)	

*All tests are executed using Wilcoxon rank-*sum test except specified*. ‡ *Kruskal*-*Wallis rank test*.

^ᵻ^*Rural units in this study are district level secondary eye units*.

Factors limiting productivity were investigated. The majority of surgeons (13, [76.5%]) responded that they could have performed more surgery if more patients had presented for treatment. These surgeons identified several key reasons for this poor patient flow. Among these are a lack of patient awareness (12, [92.3%]), cost (9, [69.2%]) and inaccessibility (6, [46.2%]). All surgeons were asked to suggest ways of developing the service, their responses included: improving community awareness (10, [59.0%]), strengthened management systems (10, [59.0%]), incentives for surgeons (7, [41.2%]) and advocacy within government (4, [23.5%]). Among the NPCS who had worked for relatively long periods, two surgeons had stopped operating during 2010 due to “a lack of support from the ophthalmologist they were working with”. Among the new NPCS graduates, one did not do any cataract surgery in 2010, as there was no operating room at the health facility they were posted to, and a further two NPCS only operated a few cases due to a “lack of support from the zonal & hospital administrators” and “not able to get appointed in time”.

### Eye care managers

Seven eye unit managers (all those available), three national NGO program coordinators and the Regional Blindness Control Program Officer were interviewed. Major themes were coded and presented with quotes in Table 4. A private clinic manager reported that there was a general shortage of supplies in the local market. The NGO program managers raised concerns over the bureaucratic challenges involved in importing surgical supplies into Ethiopia. There was also a perception that cost of surgery was high relative to patients’ ability to pay.

**Table 4:** **Results of in**-**depth interview with eye care managers**

**Discussion themes**	**Quotations**
Feeling about the service	“… *Still the service needs to be developed*; *surgeons are not using their potential*”
	“…*The service is not satisfactory*…*the backlog is still huge and attention for eye care is very poor*”
**Challenges**	
Distribution of services	“*Eye care professionals are distributed disproportionately*…*no one is willing to go to the district secondary eye units*”
	“*Cataract surgeons were trained to address the need of rural communities in responses to shortage of ophthalmologists*, *but only few were happy to work in the secondary eye units*, *and most relocate to the major towns*”
	“*Expectation of the cataract surgeons is very high*”
Poor community awareness	“*The community is not aware of the service*… *they do not know that cataract surgery service is provided regularly at their nearest health facility*…*it is always thought the service is provided by external body through campaigns*”
	“*The eye care promotion messages are not reaching to the community in need of the service*… *they just do not understand what we are talking about*… *because health promotion materials and messages are delivered in Amharic*… *not in their local language*”
Limited/no government support	“*Government officials consider eye care as vertical NGO driven program*…*they just do not have any idea how huge the problem is*…*there is no sense of ownership*”
Poor referral and reporting system	“*There is no appropriate referral system in eye care*… *the eye units are not linked both horizontally and vertically*”
	“…*There is very poor reporting system*, *cataract surgery is usually under reported*…*different institutions provide the service*… *but we rarely get the report*… *if you have to know how much is really done*, *you should go and ask every health facility*”
Surgical Supplies	“*supplies are not available in the local market*”
	“… *we have to import everything*… *the offices concerned are slow in processing the clearance*… *there are times when we are forced to transfer the supplies to other African countries*”
**Solutions**	
Community awareness	“… *Promotion of cataract surgery service should be done using local languages and appropriate media*”
	“…*Involve IECWs and local village leaders in mobilizing the service*”
	“*We should target improving the awareness of government officials*”
Community based programs	“*We need to establish comprehensive and regular outreach program*”
	“…*outreach service should be available to access the underserved communities*” “…*community based screening should be strengthened*”
Future plans to improve service	“…*establish primary eye care system and linking it with the existing secondary eye units*, *five primary eye units to one secondary eye unit*”
	“…*Improve the CSR*, *through* ‘*fast track strategy*’… *planned to perform 6000 cataract surgery per year on top of the existing number and then improving the number every year*“

A major concern among managers was the uneven distribution of services: rural communities are not well served. However, they also pointed out that the SNNPR eye care program is relatively new and still developing. One program manager explained:

“*The secondary eye units were established recently*… *many within the last three years*, *and we are hoping that more eye units will be established in other districts*.”

But there is also a feeling that building more eye units is not the only problem that needs addressing as retention and placement of surgeons and other eye care workers is difficult. Both eye unit mangers and NGO coordinators are concerned that little support has been given by the government (regional, zonal and district) in locating and retaining eye care workers:

“*Government officials do not support eye care programs*. *We (NGOs) have to pay for the training of eye care workers, establish and support the eye units and appoint and pay the surgeons… how is the program going to be sustained?*”

## Discussion

### Resource distribution

Cataract surgery units in SNNPR have reasonable resources (equipment and consumables), largely provided through NGO support. However, units and personnel were unevenly distributed. Many zones and almost half of the population had no access to services. The non-physician cataract surgeon training program was developed to help overcome the shortfall in ophthalmologists and to improve the coverage of services [17]. Although SNNPR has achieved half of its planned surgeon deployment and Vision 2020 human resource development targets, as set out by the National Vision 2020 Plan, there remains a very uneven distribution of surgeons [10,15]. This is largely attributable to the uneven distribution of eye unit locations and the unpopularity of working in more remote areas [18,19].

The reasons for the uneven distribution of staff across SNNPR are complex. Selecting the right people for training is key [17]. In Ethiopia, NPCS training is a significant career development and there is a general expectation among health workers that higher-level training leads to better placements and living condition. It is probably unrealistic to expect someone with many years experience, based in a larger town, to relocate to a rural eye unit following training. Willingness and ability to serve in district eye units should be considered in the selection process. Earlier studies have found that health workers with rural exposure or backgrounds show greater willingness to work in rural settings [19,20]. We have previously found that working and living conditions in Ethiopia are significant determinants of staff retention in a trichiasis surgery program [21]. A mechanism that rewards those who are working in rural eye units could be considered [20]. To compensate for the current inequality of service provision, regular outreach programs are needed in areas where there is little or no access to services [22].

### Surgical productivity

In 2010 the CSR for SNNPR was far below the target set in the National Vision 2020 Plan [9,15]. For individual units or surgeons, availability of equipment and consumables did not appear to be limiting factors; however, productivity was often low. The reasons for this are multiple and may include under-utilization of the service by the population and personnel management challenges (distribution, motivation and support).

No units reported a “waiting list” of patients, suggesting that either the need has been met or the community is not utilizing the service [9]. Under-utilization of this service by the community may have multiple causes: limited awareness, geographical inaccessibility, direct or indirect costs, low confidence in the service due to reported poor surgical outcomes [8,13,23]. Strategies that bridge the gap between patients and service providers need to be implemented and scaled-up in the region. A few units conducted activities designed to help bridge the gap, such as an awareness creation program, community based screening and outreach cataract surgery. These activities were associated with higher productivity in SNNPR. Moreover, other studies suggest that community-orientated approaches are needed to deliver high quality high volume cataract surgery services in low resource settings [12,22,24]. Increased support will be needed from the regional government and NGOs, as overcoming barriers to access and encouraging service utilization will require additional resources in terms of staff and logistics.

The average cataract surgeon’s productivity in SNNPR (280 cases/surgeon/year) was comparable with a report of their counterparts in four East African countries (243 cases/surgeon/year) [12]. However, there was a significant difference in output between surgeons in SNNPR. Although the data is insufficient to model, univariate analysis suggests that ophthalmologists received more financial and logistic support from NGO to conduct outreach surgery, had more experience and were more satisfied with their work environment than the NPCS. These factors were associated with higher productivity in this and other studies [12,24-26]. NPCS felt insufficiently recognized and supported by ophthalmologists and program managers. This was consistent with the findings from a recent situational analysis in SSA countries, where lack of adequate support and acceptance is leading to under-utilization of NPCS as reported by both the NPCS and their trainers [17]. In our study, some program managers also indicated that NPCSs have unmet expectations which could affect their performance. Strengthened management systems and providing training to eye care mangers of all levels, including ophthalmologists, may help to build a supportive work environment. Consistent with other studies, NPCS who received supervision tended to have greater productivity [12,25,26]. However, unfortunately most cataract surgeons in SSA do not receive regular supervision from an ophthalmologist [17]. Efforts need to be made to value the work that the NPCS are doing and provide them with equitable support if they are to succeed in addressing the cataract surgery service need in Ethiopia. Developing a system where decisions and information are shared, accountability mechanisms are strengthened and supportive supervision is provided could be transformational [26].

The aim of cataract surgery is to restore vision to an acceptable level [8]. Therefore the quality and outcomes of surgery are of central importance. Currently, outcome-monitoring mechanisms are not built into the cataract surgery program in SNNPR. Monitoring outcomes through regular audit of results is essential for quality assurance and improvement; this should be built into all cataract surgery programs [27].

#### ***Service sustainability***

The average unit cost for cataract surgery in SNNPR varied widely between health facilities. The cost of cataract surgery services in Africa is probably somewhat higher than that reported from India [28]. This is in part due to lower volumes of surgery being performed in African units and higher costs for surgical consumables [8,9]. The major determinant of the provider unit cost is the number of surgeries per year, due to numerous facility-level fixed costs [8,9,28]. Newer health facilities incurred the highest fixed equipment cost. Consistent with the literature, we found variations in the source, type, cost and procurement practices of consumables, including IOLs, and the amount of support provided by the NGOs (such as incentives) were major reasons for large variations in variable costs between eye units [9,29]. No published data are available on cataract surgery unit cost in Ethiopia.

In our study the actual cost of providing surgery was about 5 times higher than the charge to the patient in government eye units. In addition, the support from the regional government was limited to salaries and training with other resources provided by NGOs. This raises questions about the sustainability of the service in its current form [9]. In addition to increasing output, cost containment and recovery mechanisms will be needed if the service is to become more self-reliant [8,9,30].

One of the significant themes that arose from interviews with the eye care managers and surgeons is the limited government support and attention to eye care as factor limiting both productivity and service sustainability. This might be due to other competing heath care priorities, poor awareness of the extent of the problem or even the considerable NGO support to eye care [31,32]. The significant NGO support is a necessary response to the government’s limited investment in eye care. Anecdotal data shows that parts of the country without any NGO support are extremely underserved. If the Vision 2020 targets are to be achieved, cataract surgery services will need to be a health care priority for the government [30,32]. Advocacy is needed within the government for a strengthened eye care service, integrated into the health system [30-32]. The development of an integrated primary eye care program within the existing Health Extension program would help to identify and refer people needing services, boosting demand and bringing down costs [9,13,30].

This study has some limitations. An assessment of surgical outcomes in SNNPR was beyond the scope of the resources available for this study. As highlighted above, surgical outcome monitoring is a crucial component of any cataract surgery program. Cataract surgery service output is measured by the CSR; data on the number of surgeries performed on blind cases and second eyes were not collected, as this was unavailable across all eye units. This limits our study’s ability to assess the impact of the program on reducing cataract blindness. Although all parts SNNPR where cataract surgery services are provided were visited and all surgeons in the region were interviewed; the number of personnel is relatively small, limiting the scope of the analysis. Finally, the provider cost does not include building costs.

## Conclusion

Overall, the uneven distribution and under utilization of infrastructure and personnel, the limited use of the service by the population and limited government support and attention to eye care were major factors holding the service back from reaching the Vision 2020 targets in SNNPR. Addressing these issues will help improve service coverage. Community-based studies are needed to identify reasons for the limited use of the service by potential patients. Outcome monitoring systems are needed to support quality assurance and improvement.

## Abbreviations

CSR: Cataract surgical rate; ECCE: Extra capsular cataract extraction; IOL: Intraocular lens; NGO: Non-governmental organization; NPCS: Non-physician cataract surgeon; PCIOL: Posterior capsule intraocular lens; SSA: Sub-Saharan Africa; MSICS: Manual small incision cataract surgery; SNNPR: Sothern nations and nationalities peoples’ region; WHO: World health organization.

## Competing interests

The authors declare that they have no competing interests.

## Authors’ contributions

Conceived and designed the study: EH, MJB. Conducted the study: EH, ZE. Analyzed the data: EH, MJB. Wrote the first draft of the manuscript; EH. Contributed to revising the manuscript; ZE MJB. All authors’ read and approved the final manuscript.

## Pre-publication history

The pre-publication history for this paper can be accessed here:

http://www.biomedcentral.com/1472-6963/13/480/prepub

## Supplementary Material

Additional file 1 Data Record Forms. Click here for file
