# Supplementary material for: Cataract surgery in Southern Ethiopia: distribution, rates and determinants of service provision
Source: BMC Health Serv Res. 2013 Nov 19;13:480. doi: 10.1186/1472-6963-13-480 (PMC3842739; doi:10.1186/1472-6963-13-480)
Supplement: Additional file 1 — Data Record Forms. [file 1472-6963-13-480-S1.doc]

**Appendix 1 – Eye unit/ health facility data record form**

| **N** | **Tracing detail** | **Unique Study Number**   |  |  |  | | --- | --- | --- | | | |
| --- | --- | --- | --- | --- | --- | --- | --- |
| 1 | Name of the Health Facility |  | | |
| 2 | Type of health facility | 1 = District Health centre  2 = District Hospital  3 = Zonal Hospital  4 = Private/NGO clinic/Hospital  5 = Regional Hospital | |  |
| 3 | Zone |  | | |
| 4 | District |  | | |
| 5 | Catchment population covered |  | | |
| 6 | Contact person name and telephone | Name: | Tel: | |

| **N** | **Human Resource** | **Answer Options** | **Answer** |
| --- | --- | --- | --- |
| 7 | Number of Ophthalmologists | *Enter number* |  |
| 8 | Number of Cataract Surgeons | *Enter number* |  |
| 9 | Number of Ophthalmic Nurses | *Enter number* |  |
| 10 | Number of IECWs working in the department | *Enter number* |  |
| 11 | Number of trained operating theatre nurses | *Enter number* |  |
| 12 | Number of optometrists/refractionists | *Enter number* |  |
| 13 | Number of general nurses working in the eye department | *Enter number* |  |
| 14 | Number of opticians | *Enter number* |  |

| **N** | **Materials and infrastructure** | |  |  |
| --- | --- | --- | --- | --- |
| 15 | Operating room | | 0 = No  1 = Yes, separate  2 = Yes, shared |  |
| 16 | Number of beds in the hospital | | *Enter number* |  |
| 17 | Number of beds for eye department | | *Enter number* |  |
| 18 | Number of functioning operating microscope | | *Enter number* |  |
| 19 | Number of complete cataract sets | | *Enter number* |  |
| 20 | Number of operating beds | | *Enter Number* |  |
| 21 | Number of slit lamps | | *Enter number* |  |
| 22 | Keratometer | | *Enter number* |  |
| 23 | A scan | | *Enter number* |  |
| 24 | Ophthalmoscope | | *Enter number* |  |
| 25 | Indirect Ophthalmoscope | | *Enter number* |  |
| 26 | Tonometer | | *Enter number* |  |
| 27 | Trial lens set | | *Enter number* |  |
| **N** | | **Last year (2010) data** | ***Answer option*** | **Response** |
| 28 | | How many community awareness creation programmes were conducted last year? | *Write number* |  |
| 29 | How many outreach sessions performed last year (2010) | | *Write number* |  |
| 30 | How many surgeries performed in outreach in 2010 | | *Write number* |  |
| 31 | How many surgeries performed in 2010 in static programme? | | *Write number* |  |
| 32 | Total number of surgeries conducted in 2010 under the health facility? | | *Write number* |  |
| 33 | Number of patients IOL inserted in 2010 | | *Write number* |  |
| 34 | Number of people received cataract surgery by gender in 2010 | | Male |  |
|  |  |  | Female |  |
| 35 | Annual number of surgeries performed by the health facility in 2010 | | *Write number* |  |
| **N** | **Consumables and Finance** | | **Answer options** | **Answer** |
| 36 | How many days per month did you have access to complete consumables (approximately in average) | | 1 = < 15 days  2 = < 20 days  3 = < 30 days  4 = all days |  |
| 37 | How did you get your consumables? | | 0 = No consumables for cataract surgery  1 = Through hospital procurement  2 = Supplied by NGO  3 = The eye department directly buys  4 = Patients buy their own consumables from outside  5 = Donation and hospital procurement  99 = Other (describe) |  |
| 38 | How do you buy your consumables? | | 0 = Do not buy consumables  1 = Bulk purchase  2 = According to demand  3 = Twice per year  4 = Once a year  99 = Others (describe) |  |
| 39 | From whom do you buy your consumables? | | 0 = Do not buy consumables  1 = From government provider  2 = Local private provider  3 = From international provider  99 = Others (describe) |  |
| 40 | If patients buy any of the consumables which ones they buy? | | Free text |  |
| 41 | Do you mange/control the finance of the eye department | | 0 = No  1 = Yes |  |
| 42 | If yes, how much was last year’s income from cataract surgery? | | Free text |  |

| **N** | **Current Practice** | | **Answer Options** | **Answer** |
| --- | --- | --- | --- | --- |
| 43 | Does the hospital have a waiting list for cataract surgery? | | 0 = No (Go to question number 48)  1 = Yes |  |
| 44 | If there is a waiting list, how many weeks? | | Write in weeks |  |
| 45 | | Average time spent to perform a single cataract surgery during the day of health facility visit ( time between putting on and removing drape) | *Write time in minutes/NA* |  |
| 46 | | Average time gap between two cataract surgeries | *Write in minute/N/A* |  |
| 47 | | Number of surgeries performed/hour/surgeon | *Enter number* |  |
| 48 | | How did you get your cataract patients? | *0 = No surgery*  *1 = They present by themselves*  *2 = Through community workers*  *3 = By providing transportation access from village level*  *99 = Others (describe)* |  |
| 49 | | What measures are taken to increase number of cataract patients? | *Free text* |  |
| 50 | How many cataract surgery days/week are allocated? | | *Write number* |  |
| 51 | At what time actual surgery starts? | | *Enter surgery starting hour* |  |
| 52 | Average number of hours spent in a single day cataract session | | *Write number* |  |
| 53 | Average number of surgeries performed per session | | *Write number* |  |
| 54 | How many days patients stay in hospital for cataract surgery? | | *Write number* |  |
| 55 | Do you have separate sterilization system for the eye department? | | *0 = No*  *1 = Yes* |  |
| 56 | What method (s) do you use for sterilization? | | *1 = Steam sterilization*  *2 = Dry oven*  *3 = Chemical*  *4 = Steam sterilization and dry oven*  *99 = Other (describe)* |  |
| 57 | Do you have access to water all the time? | | 0 = No  1 = Yes |  |
| 58 | Do you have access to electricity supply all the time? | | 0 = No  1 = Yes |  |
| 59 | Do you have access to maintenance service for equipments? | | 0 = No  1 = Yes |  |
| 60 | How many people had received cataract surgery in May? | | Write number |  |
| 61 | How many had attended their 1 month post OP follow-up in June? | | Write number |  |
| 62 | Number of cataract surgeries 2006 | |  |  |
| 63 | Number of cataract surgeries in 2007 | |  |  |
| 64 | Number of cataract surgeries in 2008 | |  |  |
| 65 | Number of cataract surgeries in 2009 | |  |  |

**Appendix 2: Cost and price of cataract surgery**

| **N** | **Cost of Cataract surgery** | | | | | | | | | | | | | | |
| --- | --- | --- | --- | --- | --- | --- | --- | --- | --- | --- | --- | --- | --- | --- | --- |
| **A** | **Fixed running Cost** | Number | **Hours spent per week (out of 40 hours/ week) on** | | | | | | | | | | | Salary in Birr | |
| 1 | People involved in single cataract operation |  | Patient examination | | | Surgery | | | Administration | | | | |  |  |
|  |  |  | Eye patients | | Cataract patients | All surgeries | | Cataract | Whole | | Cataract service | | |  |  |
| 2 | Ophthalmologist |  |  | |  |  | |  |  | |  | | |  | |
|  |  |  |  |  |  |  |  |  |  |  |  |  |  |  |  |
| 3 | Cataract Surgeons |  |  | |  |  | |  |  | |  | | |  | |
|  |  |  |  |  |  |  |  |  |  |  |  |  |  |  |  |
| 4 | Ophthalmic Nurse/ Nurses |  |  | |  |  | |  |  | |  | | |  | |
|  |  |  |  |  |  |  |  |  |  |  |  |  |  |  |  |
|  |  |  |  |  |  |  |  |  |  |  |  |  |  |  |  |
|  |  |  |  |  |  |  |  |  |  |  |  |  |  |  |  |
|  |  |  |  |  |  |  |  |  |  |  |  |  |  |  |  |
| 5 | Optometrist/ Refractionists |  |  | |  |  | |  |  | |  | | |  | |
| 6 | Cleaner |  |  | |  |  | |  |  | |  | | |  | |
| 7 | Others (describe) |  |  | |  |  | |  |  | |  | | |  | |
| 8 | Other running costs (overhead, utility maintenance...) | | *Write cost in birr* | | | | | |  | | | | | | |
| **9** | **Total annual fixed running costs of cataract surgery** | | ***Write Cost in Birr*** | | | | | |  | | | | | | |
| **B** | **Variable cost for single operation** | | | | | | | | | | | | | | |
| 10 | Consumables | | | | *Write cost* | | | | |  | | | | | |
| 11 | IOL | | | | *Write cost* | | | | |  | | | | | |
| 12 | Medication | | | | *Write cost* | | | | |  | | | | | |
| 13 | Other consumables (describe) | | | | *Write cost* | | | | |  | | | | | |
| 14 | Food for a single patient | | | | *Write cost* | | | | |  | | | | | |
| 15 | Incentives paid for a single surgery | | | | *Write amount* | | | | | *Static* | |  | *Campaign* | |  |
| 16 | Number of surgeries performed by incentive | | | | *Write number* | | | | | *Static* | |  | *campaign* | |  |
| **17** | **Total variable cost of a single cataract surgery** | | | | ***Write price*** | | | | |  | | | | | |
| **C** | **Price (direct patient cost) of a single cataract surgery** | | | | | | | | | | | | | | |
| 18 | Pre Op examination fee | | | | *Write price* | | | | |  | | | | | |
| 19 | IOL | | | | *Write price* | | | | |  | | | | | |
| 20 | Medication | | | | *Write price* | | | | |  | | | | | |
| 21 | Food | | | | *Write price* | | | | |  | | | | | |
| 22 | Bed | | | | *Write price* | | | | |  | | | | | |
| 23 | Other (describe) | | | | *Write Price* | | | | |  | | | | | |
| **24** | **Total direct patient cost of cataract surgery** | | | | ***Write Price*** | | | | |  | | | | | |
|  |  | | |  |  | | | | | | | | | | |
| **D** | **Single cataract surgery cost** | | | Number | ***Minute spent by cadres to provide cataract surgery service*** | | | | | | | | | | |
|  | **Cadres involved** | | |  | ***Pre op examination*** | | ***Surgery*** | | ***Post op examination*** | | | | | | |
| 1 | Ophthalmologist | | |  |  | |  | |  | | | | | | |
| 2 | Cataract surgeon | | |  |  | |  | |  | | | | | | |
| 3 | Ophthalmic Nurse | | |  |  | |  | |  | | | | | | |
| 4 | Optometrist/ Refractionists | | |  |  | |  | |  | | | | | | |
| 5 | Cleaner | | |  |  | |  | |  | | | | | | |
| 6 | Others (describe) | | |  |  | |  | |  | | | | | | |
|  |  | | |  |  | |  | |  | | | | | | |
|  |  | | |  |  | |  | |  | | | | | | |

**Consumables list for a single cataract surgery**

| **N** | **Consumables** | **Unit** | **Unit amount** | **Unit price** | **Amount needed for single surgery** | **Price for single surgery** | **Remark** |
| --- | --- | --- | --- | --- | --- | --- | --- |
| 1 | P Iodine |  |  |  |  |  |  |
| 2 | Lidocaine |  |  |  |  |  |  |
| 3 | S Glove |  |  |  |  |  |  |
| 4 | Visco |  |  |  |  |  |  |
| 5 | Syringe 5 ml |  |  |  |  |  |  |
| 6 | Gauze |  |  |  |  |  |  |
| 7 | Saline/ringer |  |  |  |  |  |  |
| 8 | Plaster |  |  |  |  |  |  |
| 9 | Alcohol |  |  |  |  |  |  |
| 10 | Dexa drop |  |  |  |  |  |  |
| 11 | CAF drop |  |  |  |  |  |  |
| 12 | Mexidol |  |  |  |  |  |  |
| 13 | Genta injection |  |  |  |  |  |  |
| 14 | Dexa injection |  |  |  |  |  |  |
| 15 | Tropicamide |  |  |  |  |  |  |
| 16 | Keratom |  |  |  |  |  |  |
| 17 | Cresent |  |  |  |  |  |  |
| 18 | Syringe 10ml |  |  |  |  |  |  |
| 19 | Insuline syringe |  |  |  |  |  |  |
| 20 | Razer blade |  |  |  |  |  |  |

**Appendix 3**: Ophthalmologists and Cataract surgeons’ Questionnaire

| **N** | **Tracing details** | **Unique study number**   |  |  |  | | --- | --- | --- | |
| --- | --- | --- | --- | --- | --- |
| 1 | Zone |  |
| 2 | District |  |
| 3 | Name of Health Facility /Institution |  |

| **N** | **Background Characteristics** | **Answer Options** | **Answer** |
| --- | --- | --- | --- |
| 4 | Gender of surgeon | 1 = Male  2 = Female |  |
| 5 | Age in years | *Enter number* |  |
| 6 | Qualification | 1 = Ophthalmologist  2 = Cataract Surgeon |  |
| 7 | Type of Health Facility | 1 = District Health centre  2 = District Hospital  3 = Zonal Hospital  4 = Private/NGO clinic/Hospital  5 = Regional referral hospital  99 = Other (describe) |  |

| **N** | **Questions about Training & Current Practice** | **Answer Options** | | | **Answer** |
| --- | --- | --- | --- | --- | --- |
| 8 | How many surgeries you did during your training | *Enter number* | | |  |
| 9 | Number of months worked as a surgeon | Enter Number | | |  |
| 10 | Are you doing cataract surgery currently? | 0 = No  1 = Yes (Go to question number 17) | | |  |
| 11 | If No, what is your reason? *(Multiple answers are possible)* | 0 = Doing surgery currently  1 = No consumables  2 = No surgical sets  3 = No operating Microscope  4 = Patients are not coming  5 = No supporting staff  6 = No operating room  7 = Not ready to perform surgery  8 = No incentive  9 = Engaged in other activities (lack of time)  99 = other (describe) | | |  |
| 12 | What is the main reason | *Enter code from above* | | |  |
| 13 | Have you ever performed surgery since your training? | 0 = No  1 = Yes ( Go to question number 18) | | |  |
| 14 | If No, what is/are your reason(s) for not doing surgery since training? (Multiple answers are possible) | 0 = Did surgery  1 = No equipment  2 = No consumables  3 = No patients  4 = No supporting person  5 = No operating room  6 = Not ready to perform surgery  7 = No incentive  99 = other (describe) | | |  |
| 15 | What measure/ step did you take to perform cataract surgery? | Free text | | | |
| 16 | What were the results/responses of your measure? | Free text | | | |
| 17 | Are you satisfied with the current work environment of your facility? | 0 = No  1 = Yes | | |  |
| 18 | What problems/ challenges do you face in providing the service? | Free text | | | |
| 19 | What do you think should be done to develop the service? | Free text | | | |
| **N** | **If perform surgery** | ***Answer Options*** | | | **Answers** |
| 20 | How many cataract surgeries have you performed since your training (approximately) | *Enter number* | | |  |
| 21 | What vision cut off point do you use to perform surgery | 1 = No vision cut of point depend on situation  2 = Less than Hand Movement  3 = Less than 1/60  4 = Less than 3/60  5 = Les than 6/60  6 = Less than 6/18  99 = other (describe) | | |  |
| 22 | How do you describe the cataract surgery service delivery programme? | 1 = Fixed time table  2 = Flexible programme  99 = Other (describe) | | |  |
| 23 | What surgical technique do you use for routine standard cataract surgery? | 1 = ICCE (AC IOL)  2 = ECCE + PC IOL  3 = SICS + PC IOL  99 = other (describe) | | |  |
| 24 | How many days per week do you operate? | *Enter number of days* | | |  |
| 25 | How many surgeries do you perform in average per session | *Enter number of surgeries* | | |  |
| 26 | How many cataract surgeries in average do you perform per hour? | Enter Number | | |  |
| 27 | Do you routinely perform refraction after cataract surgery  (During discharge) | 0 = No  1 = Yes | | |  |
| 28 | Do you have incentive(s) for doing surgery? | 0 = No  1 = Yes | | |  |
| 29 | If yes, what type of incentive(s)  (More than one answer is possible) | | 0 = No incentive  1 = Money  2 = Training  3 = Holidays  99 = other (describe) |  | |

| **N** | **Last year’s (2010) data** | **Answer Options** | **Answer** |
| --- | --- | --- | --- |
| 30 | How many times you participated in cataract outreach campaigns last year | *Enter number* |  |
| 31 | How many surgeries did you perform in outreach last year | *Enter number* |  |
| 32 | Who organizes the outreach programmes? | 0 = No outreach programmes  1 = The Health Facility  2 = Supporting NGO  99 = Other (describe) |  |
| 33 | Who cover the expenses for the campaigns? | 0 = No outreach campaigns  1 = The health facility  2 = Supporting NGO (Describe)  99 = Other (describe) |  |
| 34 | How many surgeries did you perform in static programme? | *Enter number* |  |
| 35 | How many surgeries you performed totally last year (2010) | *Enter number* |  |
| 36 | What things/ conditions helped you to do your job well? | *Free text* | |
| 37 | What have you done to increase the number of surgeries? | *Free text* |  |
| 38 | Have you ever been supervised since training? (For cataract surgeons) | 0 = No  1 = Yes  77 = Not applicable |  |
| 39 | If yes how many times last year | *Enter number or NA* |  |
| 40 | What factors prevented you from doing more surgeries last year (2010)? | 0 = No reason  1 = Incomplete cataract surgery sets  2 = Incomplete consumables  3 = Engaged in other activities/ commitments (lack of time)  4 = Poor patient flow  5 = No appropriate supporting staff  6 = Poor institutional support  7 = Lack of budget to conduct outreach  99 = Other (describe) |  |
| 41 | If poor patient flow, what do you think are the reasons?  (Multiple answers are possible) | 0 = Not applicable  1 = Lack of awareness  2 = Inaccessibility of service  3 = Lack of trust on the service  4 = Fear of surgery  5 = Cost of surgery  99 = Other (describe) |  |
| 42 | What is the most important reason? | *Enter above code as appropriate* |  |
| 43 | How much of your time do you spent in cataract patient examination per week? | *Enter time spent in hour (out of 40 hours/week)* |  |
| 44 | How much of your time do you spent in cataract surgery per week? | *Enter time spent in hour (out of 40 hours/week)* |  |
| 45 | How much of your time do you spent in administration or organization of cataract surgery service per week? | *Enter time spent in hour (out of 40 hours/week)* |  |

**Appendix 4: Managers** questionnaire

| **N** | **Questions** | **Unique study number**   |  |  |  | | --- | --- | --- | | | |
| --- | --- | --- | --- | --- | --- | --- | --- |
| 1 | Name |  | | |
| 2 | Organization |  | | |
| 3 | Position in the organization |  | | |
| 3 | What is your feeling about the service? |  | | |
| 4 | What are your achievements/ or you happy with? |  | | |
| 5 | What problems did you face in providing or organizing the service? |  | | |
| 6 | What types of support are provided? |  | | |
| 7 | What should be developed? |  | | |
| 8 | Future plans to develop the service? |  | | |
|  | **Capital cost to setup cataract surgery service** | **Answer option** | **Cost** | **Number** |
| 8 | Building | *Write cost* |  | |
| 9 | Operating Microscope | *Write cost and number* |  |  |
| 10 | Cataract sets | *Write cost and number* |  |  |
| 11 | Examination equipments (slit lamps, ophthalmoscope...) | *Write cost and number* |  |  |
| 12 | Sterilization equipments | *Write cost and number* |  |  |
| 13 | Bed | *Write cost and number* |  |  |
| 14 | Table | *Write cost and number* |  |  |
| 15 | Others (describe) | *Write cost and number* |  |  |
